# Ventricular Pneumocephalus with Meningitis after Lumbar Nerve Root Block

**DOI:** 10.1155/2013/640185

**Published:** 2013-05-16

**Authors:** Shin Ahn, Young Sang Ko, Kyung Soo Lim

**Affiliations:** Department of Emergency Medicine, Asan Medical Center, University of Ulsan, College of Medicine, 388-1, Pungnap-dong, Songpa-gu, Seoul 138-736, Republic of Korea

## Abstract

Lumbar nerve root block is a common modality used in the management of radiculopathy. Its complications are rare and usually minor. Despite its low morbidity, significant acute events can occur. Pneumocephalus is an accumulation of air in the intracranial space. It indicates a violation of the dura or the presence of infection. The object of this report is to describe the case of a patient with intraventricular pneumocephalus and bacterial meningitis after lumbar nerve root block. A 70-year-old female was brought into emergency department with severe headache and vomiting which developed during her sleep. She had received lumbar nerve block for her radiculopathy one day before her presentation. Cranial computed tomography scan revealed a few hypodense lesions in her left lateral ventricle frontal horn and basal cistern indicating ventricular pneumocephalus. Five hours later, she developed sudden hearing loss. Cerebrospinal fluid analysis showed bacterial meningitis, and she was treated with high dose steroid and antibiotics. However, her impaired hearing as a sequela from meningitis was persistent, and she is still in follow-up. Intracranial complications of lumbar nerve root block including meningitis and pneumocephalus can occur and should be considered as high-risk conditions that require prompt intervention.

## 1. Introduction

Lumbar nerve root blocks are frequently employed in the management of degenerative conditions of the lumbar spine including radiculopathy. Despite a known low morbidity, significant acute events can occur. We recently accoutered a case of sudden hearing loss associated with intraventricular pneumocephalus and meningitis after lumbar nerve root block.

## 2. Case Presentation

**Figure 1:**
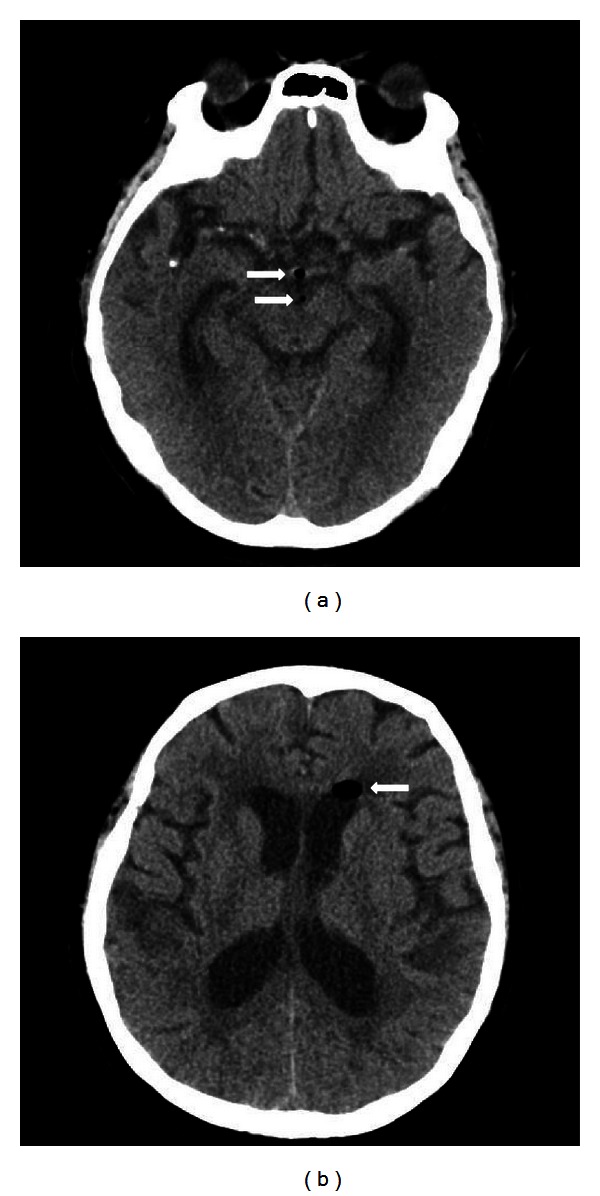
Axial computed tomography (CT) scan of head demonstrated free air (arrows) in the left lateral ventricle frontal horn and basal cistern.

 A 70-year-old female with a history of prior spine surgery (L5-S1 discectomy and posterior fusion taken 5 years ago), hypertension, and diabetes mellitus visited our emergency department with headache and vomiting. She complained of abruptly onset severe frontal headache with nausea and vomiting which developed during her sleep. She had received lumbar nerve root block for her chronic back pain a day before her presentation. She said she had no special symptoms after completion of the procedure. Her vital signs were unremarkable, and neurologic examination was normal except mild nuchal rigidity. Because of unremitting headache and vomiting, she underwent a computed tomography (CT) scan of head without contrast, which demonstrated a few hypodense lesions (−965 Hounsfield units) in her left lateral ventricle frontal horn and basal cistern (Figure 1). No other abnormal findings including intracranial bleeding, bony destruction, or sinusitis were found. Pneumocephalus was thought to be the etiology of her headache, and analgesics were given with high flow oxygen for the pneumoventricle.

 Five hours later, the patient developed an acute onset of both side hearing difficulties. Otoscopic examination was unremarkable, and to rule out meningitis, lumbar puncture for cerebrospinal fluid (CSF) was done. The local findings of her back were unremarkable. CSF analysis disclosed increased levels of total protein (219 mg/dL), leukocytosis (2650/*μ*L) with neutrophil dominance (82%), and a pressure of 21 cm H_2_O. Her diagnosis was bacterial meningitis with pneumocephalus as a complication of lumbar nerve root block. Medical treatment was established with ceftriaxone, vancomycin, and ampicillin, and after otologist consultation, high dose steroid was given for severe bilateral sensorineural hearing loss. 

 Two days later, her CSF culture revealed ampicillin susceptible *Enterococcus faecalis*. Over the next few days of treatment with methylprednisolone and antibiotics, the patient's clinical status improved except fluctuating hearing loss; after 10 days, repeated spinal fluid analysis was negative for inflammatory parameters (0 white blood cell/*μ*L), while her hearing difficulties being persistent. Once the medical therapy was completed, the patient was discharged with outpatient follow-up. 

 Ten days later, she revisited our emergency department with a newly developed vertigo. She was neurologically intact except for persisting bilateral hearing deficits. After otologic examination, diazepam was prescribed for her vertigo. At present, 3 months later, the patient is still in follow-up and shows impaired hearing as a sequela from meningitis and pneumoventricle.

## 3. Discussion

 Most cases of pneumocephalus occur as a result of discontinuity in the cranium, including skull fractures which can lead the outside air to enter the cranium through direct communication, and fracture of air-containing sinus such as the frontal, ethmoid, sphenoid, or maxillary. When air is used in the loss of resistance technique during epidural injection, pneumocephalus can be caused by air entering the meninges during the procedure, either by accidental injection or by the pressure difference created between the spine and occluded needle. Although pneumocephalus is a well-known complication of spinal and epidural anesthesia, it is extremely rare after lumbar epidural steroid injection or nerve root blocks. 

 Bacterial meningitis is also a known cause of pneumocephalus. However, most reported cases of meningitis complicating pneumocephalus are accompanied by otogenic infection with communication between the middle ear and either middle or posterior cranial fossa through bony defects [1], or pneumocephalus complicating meningitis with sinusitis [2]. Rarely, gas-forming bacterial infection could cause pneumocephalus via hematogenous spread [3]. In our case, the causative organism for meningitis was *Enterococcus faecalis*, a well-known microorganism for healthcare-associated infections. Since otoscopic findings and imaging studies showed no infectious focus and her symptoms developed abruptly on the day after nerve root block, ventricular pneumocephalus with bacterial meningitis in the present case were considered complications of nerve root block.

 The unique feature of our case is the rapid presentation of the patient's symptoms; sudden headache with vomiting and then acute onset of both sides hearing difficulties. Meningitis is a recognized cause of hearing loss. Sensorineural hearing loss tends to develop during the earliest stages of meningitis and transient or permanent sensorineural hearing losses are common in survivors from bacterial meningitis [4]. 

 Pneumocephalus has a tendency to resolve spontaneously, and usually it is absorbed after a few days. However, high inspired oxygen concentration can hasten its absorption. Increasing the inspired oxygen reduces the partial pressure of nitrogen in the blood, and by creating a greater concentration gradient for nitrogen, it fastens the intracranial air collection to diffuse into the blood stream [5].

 The present case allowed us to conclude that pneumocephalus with meningitis after lumbar nerve root block caused headache and hearing loss. Early administration of high dose steroid with antibiotics improved her symptoms. However, she had persisting bilateral hearing loss and an episode of vertigo as sequela of bacterial meningitis.

Intracranial complications of lumbar nerve root block including meningitis and pneumocephalus can occur and should be considered as high-risk conditions that require prompt intervention. 

## References

[1] Abbati SG, Torino RR (2012). Spontaneous intraparenchymal otogenic pneumocephalus: a case report and review of literature. *Surgical Neurology International*.

[2] Ohe Y, Maruyama H, Deguchi I (2012). An adult case of pneumocephalus and pneumococcal meningitis associated with the sphenoid sinusitis. *Internal Medicine*.

[3] Andrews JC, Canalis RF (1986). Otogenic pneumocephalus. *Laryngoscope*.

[4] Van De Beek D, De Gans J, Spanjaard L, Weisfelt M, Reitsma JB, Vermeulen M (2004). Clinical features and prognostic factors in adults with bacterial meningitis. *The New England Journal of Medicine*.

[5] Dexter F, Reasoner DK (1996). Theoretical assessment of normobaric oxygen therapy to treat pneumocephalus: recommendations for dose and duration of treatment. *Anesthesiology*.

